# A Student’s t Mixture Probability Hypothesis Density Filter for Multi-Target Tracking with Outliers

**DOI:** 10.3390/s18041095

**Published:** 2018-04-04

**Authors:** Zhuowei Liu, Shuxin Chen, Hao Wu, Renke He, Lin Hao

**Affiliations:** 1 Information and Navigation College Air Force Engineering University, Xi’an 710077, China; lzwlovef1@163.com (Z.L.); chenshuxin68@163.com (S.C.); lnzrds@163.com (R.H.); 2 Unit 93786, Chinese People’s Liberation Army (PLA), Zhangjiakou 075000, China; dearvic@126.com

**Keywords:** multi-target tracking, PHD filter, Student’s t mixture, outliers, robustness

## Abstract

In multi-target tracking, the outliers-corrupted process and measurement noises can reduce the performance of the probability hypothesis density (PHD) filter severely. To solve the problem, this paper proposed a novel PHD filter, called Student’s t mixture PHD (STM-PHD) filter. The proposed filter models the heavy-tailed process noise and measurement noise as a Student’s t distribution as well as approximates the multi-target intensity as a mixture of Student’s t components to be propagated in time. Then, a closed PHD recursion is obtained based on Student’s t approximation. Our approach can make full use of the heavy-tailed characteristic of a Student’s t distribution to handle the situations with heavy-tailed process and the measurement noises. The simulation results verify that the proposed filter can overcome the negative effect generated by outliers and maintain a good tracking accuracy in the simultaneous presence of process and measurement outliers.

## 1. Introduction

Multi-target tracking (MTT) plays an important role in many sensing systems, such as infrared, radar, sonar, etc., which uses the sensor data to jointly estimate the target state and the number of targets. Nowadays it is widely used in civilian and military applications such as air traffic control, remote sensing, ballistic missile guidance, and computer vision [1,2]. In MTT, the time-varying number of targets causes a problem in that the associations between state and measurement sets of targets are hard to know, which makes the traditional data association-based multi-target tracking methods problematic. In recent years, the random finite set (RFS) theory-based multi-target tracking filters, such as probability hypothesis density (PHD) filter [3], cardinalized PHD (CPHD) filter [4], multi-target multi-Bernoulli (MeMber) filter [1] and cardinality-balanced MeMBer (CBMeMBer) filter [5], have attracted much more attention since they can avoid the combinatorial problem that arises from data association. Moreover, some labeled RFS-based multi-Bernoulli filters [6,7] which can accommodate target tracks were proposed.

The focus of this paper is the PHD filter, which has relatively simple recursion, making it suitable for the applications demanding real time results. The PHD filter provides a tractable sub-optimal strategy for jointly estimating the number and the state of a variable number of targets by propagating the first-order statistical moment of multi-target posterior probability density in time. It has two basic implementations: the sequential Monte Carlo (SMC) method [8] and the Gaussian mixture (GM) method [9] which can solve the problem of computationally intractable multiple integrals involved in the PHD recursions. Compared to SMC implementation, GM implementation of the PHD filter has the advantages of simple state extraction and low computational cost, which is suitable for the requirement of real-time scenes. Moreover, some nonlinear extensions [9,10,11] and improvements [12,13,14] extend the scope of applications for the GM-PHD filter.

In MTT, noise, as the important part of measurement uncertainty, is an inevitable problem that reduces estimation accuracy of the PHD filter. Vo [15] thought that the setting of a reasonably large noise variance can accommodate noise interference in most situations. However, this method is only suitable for Gaussian noise. In real applications, it is hard for the measurement noise from sensor data to follow the Gaussian distribution because of electromagnetic interference or sensors’ own unreliability. Such measurements with outliers, which often express heavy-tailed character, degrade the performance of the PHD filter strikingly. What’s worse, in some real applications, such as tracking some agile targets with unreliable sensors, outliers may appear in not only the measurement model, but also the process model. This situation with simultaneous heavy-tailed process and measurement noises reduces the performance of the PHD filter severely, and can even make it break down. Although the SMC-PHD filter can deal with the problem to a certain degree, it has to pay a high computational cost, especially in high dimensions. For the GM-PHD filter, its foundational Gaussian approximation limits the capability to handle heavy-tailed non-Gaussian noises. Although Huber’s M-estimation theory [16] or variational Bayesian (VB) method [17] can be utilized to improve the performance of the GM-PHD filter against outliers in the measurement model, they both cannot handle the outliers in the process model. More importantly, the two methods above do not change the foundation of the Gaussian approximation-based GM-PHD filter. This means that the noise model still cannot match the outliers-corrupted process and measurement noises well, leading to biased estimates of the target state and the number of targets. Obviously, the Gaussian noise model cannot handle the heavy-tailed non-Gaussian noise, so how to model the heavy-tailed noise becomes the key point. As [18] said, Student’s t distribution, which has a heavy tail characteristic, is a good choice to match the heavy-tailed non-Gaussian noise. Under the Bayesian filtering framework, the Student’s t approximation-based closed form recursions are obtained for the linear system [18]. Further, the Student’s t approximation-based approach also can be used in the nonlinear system [19,20,21,22]. However, up to the present, Student’s t approximation-based approaches to approximate a PHD filter with heavy-tailed process and measurement noises do not exist.

In this paper, a novel implementation of the PHD filter is proposed based on Student’s t mixture approximation, intending to improve the estimation accuracy in terms of the target states and the target number in the presence of heavy-tailed process and measurement noises. The proposed approach models the process noise and the measurement noise as a Student’s t distribution, meanwhile, the multi-target prior intensity is approximated as a mixture of the Student’s t distributions. Then, the Student’s t mixture-approximated predicted intensity and posterior intensity are obtained through utilizing Student’s t approximation, forming a closed form recursion of the PHD filter. The Student’s t mixture implementation is proposed in RFS-based MTT algorithms for the first time. Compared to the GM case, it is a Student’s t-based implementation, which propagates a mixture of Student’s t components. Because it utilizes the heavy-tailed characteristic of Student’s t distribution, the proposed filter has better accuracy and robustness in MTT scenes with heavy-tailed process and measurement noises. Moreover, the proposed filter also has relatively low computing cost just like the GM-PHD filter. The above advantages of the proposed approach are verified by simulations designed in linear scenario and nonlinear scenario, respectively.

The remainder of this paper is organized as follows: Section 2 presents an overview of the PHD filter and some properties of the Student’s t distribution. Section 3 presents the proposed filter for linear system and extends the proposed filter to the nonlinear system. Simulation results are given in Section 4, and conclusions are drawn in Section 5.

## 2. Background

### 2.1. The PHD Filter

The random finite set (RFS) approach [1] provides a mathematically elegant treatment for the difficult problem that is how to estimate the time-varying number of targets and their states jointly in MTT scenarios. According to RFS theory, the collections of target states and measurements at time *k* can be represented as finite sets $X_{k}=\{x_{k,1},\ldots ,x_{k,M(k)}\}\in F(X)$ ($X\subseteq {\mathbb{R}}^{d_{x}}$) and $Z_{k}=\{z_{k,1},\ldots ,z_{k,N(k)}\}\in F(Z)$ ($Z\subseteq {\mathbb{R}}^{d_{z}}$) where M(k) and N(k) are the number of targets and the number of measurements, respectively, F(X) and F(Z) are the collections of all finite subsets of target states and measurements, respectively, $d_{x}$ and $d_{z}$ are the dimensions of the state space X and the measurement space Z, respectively. Let:

(1) $$X_{k}=\left[\underset{{}_{x\in X_{k-1}}}{\cup }S_{k|k-1}(x)\right]\cup \left[\underset{{}_{x\in X_{k-1}}}{\cup }B_{k|k-1}(x)\right]\cup \Gamma_{k},$$

where $S_{k|k-1}(x)$ is the RFS of survival targets at time *k* from target states $X_{k-1}$ and $B_{k|k-1}(x)$ is the RFS of spawned targets at time k from target states $X_{k-1}$, and $\Gamma_{k}$ is the RFS of birth targets at time *k*.

The RFS of measurements $Z_{k}$ is defined as:

(2) $$Z_{k}=\left[\underset{{}_{x\in X_{k}}}{\cup }G_{k}(x)\right]\cup K_{k},$$

where $G_{k}(x)$ and $K_{k}$ are the RFS of measurements coming from target and clutter at time *k*, respectively.

Based on the RFS approach, the MTT problem was recast into a Bayesian filtering framework, but optimal Bayesian recursions are computationally intractable due to their sets integral. Then the PHD filter, which recursively propagates the first-order moment of multi-target posterior probability density, was proposed as an approximate solution in [3]. Let $D_{k-1}(x)$ denote the posterior intensity function at time *k* − 1, then the prediction step of the PHD filter is given by

(3) $$D_{k|k-1}(x)=\int \left(p_{s,k}(u)f_{k|k-1}(x|u)+\beta_{k|k-1}(x|u))D_{k-1}(u\right)du+\gamma_{k}(x),$$

where $f_{k|k-1}(•|•)$ is transition density function in a Markov process from time *k* − 1 to time *k*, $p_{S,k}(x)$ is the survival probability of each target at time *k*, $\beta_{k|k-1}(x|u)$ is the intensity function of the RFS $B_{k|k-1}(x|u)$ of targets spawning from previous state *u*, $\gamma_{k}(x)$ is the intensity function of birth targets at time *k*.

Given the predicted intensity $D_{k|k-1}(x)$, the update intensity $D_{k}(x)$ can be given by:

(4) $$D_{k}(x)=[1-p_{D,k}(x)+\sum_{z\in Z_{k}}\frac{p_{D}(x)g_{k}\left(z|x\right)}{\kappa_{k}(z)+\int p_{D}(u)g_{k}\left(z|u\right)D_{k|k-1}(u)du}]D_{k|k-1}(x),$$

where $p_{D,k}(x)$ is the probability of detection at time *k*, $g_{k}\left(z|x\right)$ denotes the measurement likelihood function at time *k*, $\kappa_{k}(x)$ is the intensity function of clutter. The integration domain of the integral functions in (3) and (4) is the state space $X\subseteq {\mathbb{R}}^{d_{x}}$.

### 2.2. Review of the GM-PHD Filter

The GM-PHD filter approximates the intensity function of multi-target as Gaussian mixture components under the assumption of the linear Gaussian dynamical model and measurement model. In addition, it still needs to satisfy the following assumptions: (1) the survival and detection probabilities are state-independent; (2) the intensities of the birth and spawn RFSs are Gaussian mixtures of the form. Then a closed-form solution of the PHD filter can be obtained as follows.

Prediction: The Gaussian mixture formulation of the PHD recursion at time *k* − 1 is given as:

(5) $$D_{k-1}(x)=\sum_{j=1}^{J_{k-1}}w_{k-1}^{\left(j\right)}N(x;m_{k-1}^{\left(j\right)},P_{k-1}^{\left(j\right)}),$$

where N(x;•,•) denotes that random vector *x* follows Gaussian distribution, $m_{k-1}^{\left(j\right)}$ and $P_{k-1}^{\left(j\right)}$ are the mean and the covariance matrix of the *j*th item in all Gaussian mixture components, respectively, $w_{k-1}^{\left(j\right)}$ is the corresponding weight, $J_{k-1}^{}$ is the number of Gaussian components.

Assume that the intensities of survival targets, spawned targets and birth targets are Gaussian mixtures of the form, the predicted intensity $D_{k|k-1}(x)$ is given by:

(6) $$D_{k|k-1}(x)=\sum_{j=1}^{J_{k|k-1}}w_{k|k-1}^{\left(j\right)}N(x;m_{k|k-1}^{\left(j\right)},P_{k|k-1}^{\left(j\right)}),$$

where:

(7) $$w_{k|k-1}^{\left(j\right)}=p_{S,k}w_{k-1}^{\left(j\right)},$$

(8) $$m_{k|k-1}^{\left(j\right)}=F_{k-1}m_{k-1}^{\left(j\right)},$$

(9) $$P_{k|k-1}^{\left(j\right)}=F_{k-1}P_{k-1}^{\left(j\right)}F_{k-1}^{T}+Q_{k-1}.$$

Update: Given the PHD predictor as Equation (6), the PHD updater is formulated as:

(10) $$D_{k}(x)=(1-p_{D,k})D_{k|k-1}(x)+\sum_{z\in Z_{k}}\sum_{j=1}^{J_{k|k-1}}w_{k}^{\left(j\right)}(z)N(x;m_{k}^{\left(j\right)}(z),P_{k}^{\left(j\right)}(z)),$$

where:

(11) $$w_{k}^{\left(j\right)}(z)=\frac{p_{D,k}w_{k|k-1}^{\left(j\right)}q_{k}^{\left(j\right)}\left(z\right)}{\kappa_{k}(z)+p_{D,k}\sum_{l=1}^{J_{k|k-1}}w_{k|k-1}^{\left(l\right)}q_{k}^{\left(l\right)}\left(z\right)},$$

(12) $$q_{k}^{\left(j\right)}\left(z\right)=N\left(z;H_{k}m_{k|k-1}^{\left(j\right)},H_{k}P_{k|k-1}^{\left(j\right)}H_{k}^{T}+R_{k}\right),$$

(13) $$m_{k}^{\left(j\right)}(z)=m_{k|k-1}^{\left(j\right)}+K_{k}^{\left(j\right)}(z-H_{k}m_{k|k-1}^{\left(j\right)}),$$

(14) $$P_{k}^{\left(j\right)}=P_{k|k-1}^{\left(j\right)}-K_{k}^{\left(j\right)}H_{k}P_{k|k-1}^{\left(j\right)},$$

(15) $$K_{k}^{\left(j\right)}=P_{xz,k|k-1}^{\left(j\right)}{\left(P_{zz,k|k-1}^{\left(j\right)}\right)}^{-1},$$

in the above, $F_{k-1}^{}$ and $H_{k}^{}$ are the state transition matrix and observation matrix, respectively, $Q_{k-1}^{}$ and $R_{k}^{}$ are the process and measurement noise covariance, respectively.

### 2.3. Student’s t Distribution

Let a random vector $x\in {\mathbb{R}}^{d}$ admit the Student’s t distribution; its probability density function (PDF) can then be expressed as [23]:

(16) $$p\left(x\right)=\frac{\Gamma (\frac{\upsilon +2}{2})}{\Gamma (\frac{\upsilon }{2})}\frac{1}{{\left(\upsilon \pi \right)}^{d/2}}\frac{1}{\sqrt{\det\left(P\right)}}{\left(1+\frac{\Delta^{2}}{\upsilon }\right)}^{-(\frac{\upsilon +2}{2})},$$

where Γ(•) denotes Gamma function and $\Delta^{2}={\left(x-m\right)}^{T}P^{-1}(x-m)$. The above PDF abbreviated by St(x;m,P,υ) with mean *m*, scale matrix *P* and degrees of freedom parameter υ. The corresponding covariance matrix can be calculated as $\frac{\upsilon }{\upsilon -2}P$ (υ>2) [23].

The tail behavior of the Student’s t distribution is very much influenced by the degrees of freedom parameter υ. The smaller υ is, the heavier the tail is, and vice versa. In addition, the Student’s t distribution reduces to the Gaussian as υ tends to infinity, thus includes it as a special case. A number of convenient properties are shared by both and can be easily derived. As for the PDF of affine mappings of Student’s t variables [23] we have that z=Ax+b, with appropriate *A* and *b*, admits:

(17) $$p\left(z\right)=St\left(z;Am+b,APA^{T},\upsilon \right),$$

The degrees of freedom parameter remains unaltered. Turning to random vectors $x_{1}\in {\mathbb{R}}^{d_{1}}$ and $x_{2}\in {\mathbb{R}}^{d_{2}}$ that are jointly Student’s t distributed with:

(18) $$p\left(x_{1},x_{2}\right)=St\left(\begin{array}{cccc} \left[\begin{array}{c} x_{1}\\ x_{2} \end{array}\right]; & \left[\begin{array}{c} m_{1}\\ m_{2} \end{array}\right], & \left[\begin{array}{c} \begin{array}{cc} P_{11} & P_{12} \end{array}\\ \begin{array}{cc} P_{12}^{T} & P_{22} \end{array} \end{array}\right], & \upsilon_{} \end{array}\right),$$

The marginal PDF of $x_{1}$ can be computed by applying a linear transformation with A=[I      O] to (18):

(19) $$p\left(x_{1}\right)=St\left(x_{1};m_{1},P_{11},\upsilon \right)$$

From (18) and (19), the conditional PDF is also a Student’s t as follows:

(20) $$p\left(x_{1}|x_{2}\right)=St\left(x_{1|2};m_{1|2},P_{1|2},\upsilon_{1|2}\right)$$

with:

(21) $$m_{1|2}=m_{1}+P_{12}P_{22}^{-1}(x_{2}-m_{2}),$$

(22) $$P_{1|2}=\frac{\upsilon_{}+\Delta_{2}^{2}}{\upsilon_{}+d_{2}}(P_{11}-P_{12}P_{22}^{-1}P_{12}^{T}),$$

(23) $$\upsilon_{1|2}=\upsilon_{}+d_{2},$$

(24) $$\Delta_{2}^{2}={\left(x_{2}-m_{2}\right)}^{T}P_{22}^{-1}(x_{2}-m_{2}),$$

It can be seen that the conditional mean (21) corresponds to the Gaussian conditional mean. The matrix parameter (22) is a scaled version of the conditional covariance in the Gaussian case, which is recovered as υ tends to infinity. In contrast to the Gaussian, $P_{1|2}$ depends on $x_{2}$. Also, the degrees of freedom parameter in (23) increases. Above properties of Student’s t distribution like (17) and (20) are the foundations of Lemmas 1 and 2 (in Section 3.3) that are the key of our proposed approach.

## 3. Student’s t Mixture PHD Recursion

Differing from the GM-PHD recursion, the proposed approach approximates the intensity of multi-target RFS as the Student’s t mixture of the form, which is propagated in the closed-form recursions. The following gives the novel algorithm for linear system at first and then extends it to nonlinear system.

### 3.1. Basic Assumptions for Linear Model

As for the linear system, some foundational assumptions are given as follows.

**Assumption** **1.** *Given that the process and measurement noises admit the zero-mean t distribution with scale matrix $Q_{k-1}^{}$ and $R_{k}^{}$, respectively, and the initial state vector also follows a t distribution, each target follows a linear Student’s t dynamical model and the sensor has a linear Student’s t measurement model, i.e.:*

(25) $$f_{k|k-1}^{}\left(x|\zeta \right)=St\left(x;F_{k-1}^{}\zeta ,Q_{k-1}^{},\upsilon_{1}\right),$$

(26) $$g_{k}^{}\left(z|x\right)=St\left(z;H_{k}^{}x,R_{k}^{},\upsilon_{2}\right),$$

*where $F_{k-1}^{}$ and $H_{k}^{}$ denote the transition matrix and measurement matrix, respectively. And assume the matrices $F_{k-1}^{}$, $H_{k}^{}$, $Q_{k-1}^{}$ and $R_{k}^{}$ are known.*

**Assumption** **2.** *The survival and detection probabilities are state independent, i.e.:*

(27) $$p_{S,k}(x)=p_{S,k},$$

(28) $$p_{D,k}(x)=p_{D,k}.$$

**Assumption** **3.** *The intensities of the birth and spawn RFSs are Student’s t mixtures of the form:*

(29) $$\gamma_{k}^{}\left(x\right)=\sum_{i=1}^{J_{\gamma ,k}}w_{\gamma ,k}^{\left(i\right)}St\left(x;m_{\gamma ,k}^{\left(i\right)},P_{\gamma ,k}^{},\upsilon_{3}\right),$$

(30) $$\beta_{k|k-1}^{}\left(x|\zeta \right)=\sum_{j=1}^{J_{\beta ,k}}w_{\beta ,k}^{\left(j\right)}St\left(x;F_{\beta ,k-1}^{\left(j\right)}\zeta +d_{\beta ,k-1}^{\left(j\right)},Q_{\beta ,k-1}^{\left(j\right)},\upsilon_{3}\right).$$

**Figure 1:**
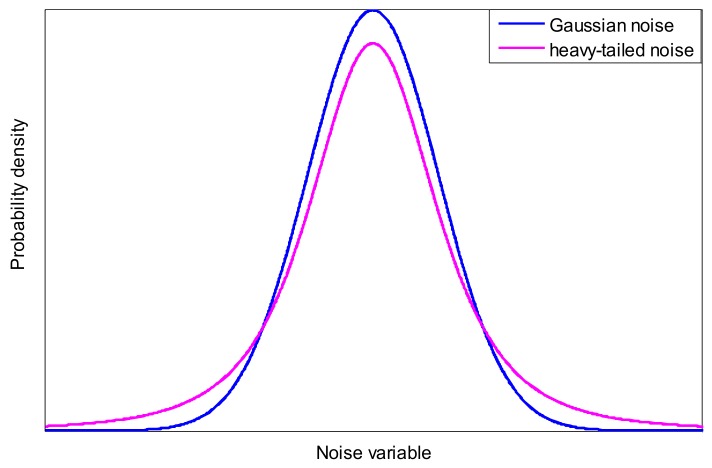
Illustration of heavy-tailed noise distribution and Gaussian noise distribution.

Assumption 1 is given according the affine transition nature of Student’s t variables [19]. Assumption 2 is commonly used in GM-PHD filters. Assumption 3 is given in the presence of process and measurement noises with heavy tails, so it is reasonable in actual applications, i.e., when using unreliable sensors or sensors suffer from electromagnetic interference while tracking some agile targets, where both process noise and measurement noise are prone to show a heavy-tailed character. For illustrating this, a one-dimensional heavy-tailed noise distribution and Gaussian noise distribution are given as Figure 1.

### 3.2. Student’s t Mixture PHD Recursion

Based on the Assumptions 1–3, the closed solution to the PHD recursion (3) and (4) is presented as two Propositions. The proposed Propositions show how the Student’s t components of the posterior intensity are analytically propagated to the next time, analogous to the GM-PHD recursion.

**Proposition** **1.** *Suppose that Assumptions 1–3 hold and that the posterior intensity at time k − 1 is a Student’s t mixture of the form:*

(31) $$D_{k-1}(x)=\sum_{j=1}^{J_{k-1}}w_{k-1}^{\left(j\right)}St(x;m_{k-1}^{\left(j\right)},P_{k-1}^{\left(j\right)},\upsilon_{3}),$$

*Then, the predicted intensity at time k is also a Student’s t mixture and is given by:*

(32) $$D_{k|k-1}(x)=D_{S,k|k-1}(x)+D_{\beta ,k|k-1}(x)+\gamma_{k}^{}\left(x\right),$$

*where:*

(33) $$D_{S,k|k-1}(x)=p_{S,k}\sum_{j=1}^{J_{k-1}}w_{k-1}^{\left(j\right)}St(x;m_{S,k|k-1}^{\left(j\right)},P_{S,k|k-1}^{\left(j\right)},\upsilon_{3}),$$

(34) $$m_{S,k|k-1}^{\left(j\right)}=F_{k-1}m_{k-1}^{\left(j\right)},$$

(35) $$P_{S,k|k-1}^{\left(j\right)}=F_{k-1}P_{k-1}^{\left(j\right)}F_{k-1}^{T}+\frac{\upsilon_{3}-2}{\upsilon_{3}}\frac{\upsilon_{1}}{\upsilon_{1}-2}Q_{k-1},$$

(36) $$D_{\beta ,k|k-1}(x)=\sum_{j=1}^{J_{k-1}}\sum_{l=1}^{J_{\beta ,k}}w_{k-1}^{\left(j\right)}w_{\beta ,k}^{\left(l\right)}St(x;m_{\beta ,k|k-1}^{\left(j,l\right)},P_{\beta ,k|k-1}^{\left(j,l\right)},\upsilon_{3}),$$

(37) $$m_{\beta ,k|k-1}^{\left(j,l\right)}=F_{\beta ,k-1}^{\left(l\right)}m_{k-1}^{\left(j\right)}+d_{\beta ,k-1}^{\left(l\right)},$$

(38) $$P_{\beta ,k|k-1}^{\left(j,l\right)}=F_{\beta ,k-1}^{\left(l\right)}P_{\beta ,k-1}^{\left(j\right)}{\left(F_{\beta ,k-1}^{\left(l\right)}\right)}^{T}+\frac{\upsilon_{3}-2}{\upsilon_{3}}\frac{\upsilon_{1}}{\upsilon_{1}-2}Q_{\beta ,k-1}.$$

**Proposition** **2.** *Suppose that Assumptions 1–3 hold and that the predicted intensity for time k is a Student’s t mixture of the form:*

(39) $$D_{k|k-1}(x)=\sum_{j=1}^{J_{k|k-1}}w_{k|k-1}^{\left(j\right)}St(x;m_{k|k-1}^{\left(j\right)},P_{k|k-1}^{\left(j\right)},\upsilon_{3}),$$

*Then, the posterior intensity at time k is also a Student’s t mixture and is given by:*

(40) $$D_{k}(x)=(1-p_{D,k})D_{k|k-1}(x)+\sum_{z\in Z_{k}}D_{k}(x;z),$$

*where:*

(41) $$D_{k}(x;z)=\sum_{j=1}^{J_{k|k-1}}w_{k}^{\left(j\right)}(z)St(x;m_{k|k}^{\left(j\right)}\prime (z),P_{k|k}^{\left(j\right)}\prime (z),\upsilon_{3}\prime ),$$

(42) $$w_{k}^{\left(j\right)}(z)=\frac{p_{D,k}w_{k|k-1}^{\left(j\right)}q_{k}^{\left(j\right)}\left(z\right)}{\kappa_{k}(z)+p_{D,k}\sum_{l=1}^{J_{k|k-1}}w_{k|k-1}^{\left(l\right)}q_{k}^{\left(l\right)}\left(z\right)},$$

(43) $$m_{k|k}^{\left(j\right)}\prime (z)=m_{k|k-1}^{\left(j\right)}+K_{k}^{\left(j\right)}(z-H_{k}m_{k|k-1}^{\left(j\right)}),$$

(44) $$P_{k|k}^{\left(j\right)}\prime (z)=\frac{\upsilon_{3}+\Delta^{2}}{\upsilon_{3}+d_{z}}(P_{k|k-1}^{\left(j\right)}-K_{k}^{\left(j\right)}H_{k}P_{k|k-1}^{\left(j\right)}),$$

(45) $$\upsilon_{3}\prime =\upsilon_{3}+d_{z},$$

(46) $$q_{k}^{\left(j\right)}\left(z\right)=St\left(z;H_{k}m_{k|k-1}^{\left(j\right)},P_{zz,k|k-1}^{\left(j\right)},\upsilon_{3}\right),$$

(47) $$K_{k}^{\left(j\right)}=P_{xz,k|k-1}^{\left(j\right)}{\left(P_{zz,k|k-1}^{\left(j\right)}\right)}^{-1},$$

(48) $$\Delta^{2}={\left(z-H_{k}m_{k|k-1}^{\left(j\right)}\right)}^{T}{\left(P_{zz,k|k-1}^{\left(j\right)}\right)}^{-1}(z-H_{k}m_{k|k-1}^{\left(j\right)}),$$

(49) $$P_{zz,k|k-1}^{\left(j\right)}=H_{k}P_{k|k-1}^{\left(j\right)}H_{k}^{T}+\frac{\upsilon_{3}-2}{\upsilon_{3}}\frac{\upsilon_{2}}{\upsilon_{2}-2}R_{k},$$

(50) $$P_{xz,k|k-1}^{\left(j\right)}=P_{k|k-1}^{\left(j\right)}H_{k}^{T}.$$

Aforementioned Propositions 1 and 2 can be established by applying the following approximated results for Student’s t functions.

**Lemma** **1.** *Given that the jointly PDF of the current state and one-step ahead state vectors is Student’s t and F, Q, m and P of appropriate dimensions and that Q and P are positive definite:*

(51) $$\int St(x;F\zeta ,Q,\upsilon_{1})St(\zeta ;m,P,\upsilon_{3})d\zeta =St(x;Fm,FPF^{T}+\frac{(\upsilon_{3}-2)\upsilon_{1}}{(\upsilon_{1}-2)\upsilon_{3}}Q,\upsilon_{3}).$$

**Lemma** **2.** *Given that the jointly PDF of the state and measurement vectors is Student’s t and H, R, m, P of appropriate dimensions and that R and P are positive definite:*

(52) $$St(z;Hx,R,\upsilon_{2})St(x;m,P,\upsilon_{3})=q(z)St(x;m\prime ,P\prime ,\upsilon_{3}\prime ),$$

*where:*

(53) $$q(z)=St(z;Hm,HPH^{T}+\frac{(\upsilon_{3}-2)\upsilon_{2}}{(\upsilon_{2}-2)\upsilon_{3}}R,\upsilon_{3}),$$

(54) m′=m+K(z−Hm),

(55) $$P\prime =\frac{\upsilon_{3}+\Delta^{2}}{\upsilon_{3}+d_{z}}(P-KHP),$$

(56) $$\upsilon_{3}\prime =\upsilon_{3}+d_{z},$$

(57) $$K=PH^{T}\left(HPH^{T}+\frac{(\upsilon_{3}-2)\upsilon_{2}}{(\upsilon_{2}-2)\upsilon_{3}}R\right){}^{-1},$$

(58) $$\Delta^{2}={\left(z-Hm\right)}^{T}\left(HPH^{T}+\frac{(\upsilon_{3}-2)\upsilon_{2}}{(\upsilon_{2}-2)\upsilon_{3}}R\right){}^{-1}(z-Hm).$$

Lemmas 1 and 2 are derived based on the properties of Student’s t distribution listed in Section 2.3. The detailed derivations of Lemmas 1 and 2 can be seen [19].

Proposition 1 is established by substituting (25), (27) and (29)–(31) into the PHD prediction (3), and replacing integrals of the form (51) by appropriate Student’s t as given by Lemma 1. Similarly, Proposition 2 is established by substituting (26), (28) and (39) into the PHD update (4), and then replacing integrals of the form (51) and product of Student’s t of the form (52) by appropriate Student’s t as given by Lemmas 1 and 2 respectively. The concrete proofs of Propositions 1 and 2 are given in Appendix B.

From (45), we know that the degree of freedom $\upsilon_{3}\prime$ will increase infinitely with recursion performing, which results in that the Student’s t mixture degrades the Gaussian mixture according to [18]. It means that the robustness against outliers for the Student’s t mixture PHD filter will be lost with time going by. To solve the problem, we adapts the moment matching approach [18] to obtain the correction of posterior intensity at time *k* given as (59)–(61). A pseudocode of main process of the proposed algorithm is given by Table A1 in Appendix A:

(59) $$D_{k}(x;z)=\sum_{j=1}^{J_{k}}w_{k}^{\left(j\right)}(z)St(x;m_{k|k}^{\left(j\right)}(z),P_{k|k}^{\left(j\right)}(z),\upsilon_{3}),$$

(60) $$m_{k|k}^{\left(j\right)}(z)=m_{k|k}^{\left(j\right)}\prime (z),$$

(61) $$P_{k|k}^{\left(j\right)}(z)=\frac{\upsilon_{3}-2}{\upsilon_{3}}\frac{\upsilon_{3}\prime }{\upsilon_{3}\prime -2}P_{k|k}^{\left(j\right)}\prime (z).$$

**Remark** **1.** *The degree of freedom parameters* $\upsilon_{1},\upsilon_{2},\upsilon_{3}$ *for the process noise model, measurement noise model and the initial multi-target state intensity are different in general. As in the recursion performed from (31) to (39), there is a problem that how to select the degree of freedom parameter between* $\upsilon_{1}$ *and* $\upsilon_{3}$ *as the degree of freedom parameter of predicted multi-target intensity to be propagated in the next recursive step. A valid method* [18] *is that choose the minimized value between* $\upsilon_{1}$ *and* $\upsilon_{3}$ *to be propagated. The same problem, existing in the recursion from (39) to (40), also can be handled by selecting the minimized value between* $\upsilon_{2}$ *and* $\upsilon_{3}$*. For simplicity, this paper assumes that the degree of freedom parameters* $\upsilon_{1},\upsilon_{2},\upsilon_{3}$ *are equal.*

**Remark** **2.** Analogous to the derivation of GM implementation of the CPHD filter in [24], the proposed Student’s t mixture implementation shown as in Propositions 1 and 2 can also be used in the CPHD filter, forming the corresponding Student’s t mixture CPHD recursion.

### 3.3. Implementation Issues

Like the GM-PHD filter, the Student’s t mixture PHD (STM-PHD) filter also suffers from the computation problem that the number of Student’s t components increases endlessly with recursive time, so a pruning procedure and a merging procedure are necessary for the STM-PHD filter. The concrete procedures are similar to the procedures in the GM-PHD filter (readers can refer to [9]). The different point is that the Student’s t components give the scale matrix not the covariance matrix, so the covariance matrix, calculated by $\frac{\upsilon }{\upsilon -2}P$, should be used in the merging procedure of the STM-PHD recursion. The estimated number of targets is obtained by summing up the weights of all the Student’s t components. This procedure for the STM-PHD filter is no different from the GM-PHD filter. Again, the state extraction is also analogous to the GM-PHD filter as to select the means of the Student’s t components that have weights greater than some threshold (generally set as 0.5 [9]).

In addition, for the scene with high clutter density, gating strategy is always used to reduce the computing cost for the GM-PHD filter. It is easy to know that the gating strategy is also suitable for the STM-PHD filter. The core principle to select the measurement can be expressed as:

(62) $$z_{k}^{\left(i\right)}\{\begin{array}{l} \in {\tilde{Z}}_{k}^{}\;\mathrm{if}\;\exists (i,j)|{\left(z_{k}^{\left(i\right)}-H_{k}m_{k|k-1}^{\left(j\right)}\right)}^{T}{\left(P_{zz,k|k-1}^{\left(j\right)}\right)}^{-1}(z_{k}^{\left(i\right)}-H_{k}m_{k|k-1}^{\left(j\right)})\leq T\\ \notin {\tilde{Z}}_{k}^{}\;\mathrm{otherwise}. \end{array}$$

where ${\tilde{Z}}_{k}^{}$ denotes the reduced set of the measurement at time *k*, $P_{zz,k|k-1}^{\left(j\right)}$ is the innovation covariance matrix corresponding to the *j*th predicted measurement, and T is the gate threshold. In contrast to the Gaussian mixture case, the judging variable ${\left(z_{k}^{\left(i\right)}-H_{k}m_{k|k-1}^{\left(j\right)}\right)}^{T}{\left(P_{zz,k|k-1}^{\left(j\right)}\right)}^{-1}(z_{k}^{\left(i\right)}-H_{k}m_{k|k-1}^{\left(j\right)})$ for the Student’s t mixture case follows an F-distribution not a chi-squared distribution. It means that the gate threshold for the STM-PHD filter, which can be chosen from F-distribution table, is different from the GM-PHD filter at the same probability regions.

### 3.4. Extension to Nonlinear Model

This section considers the situation that process and measurement models are nonlinear. The models in Assumption 1 change to become:

(63) $$x_{k}=f_{k}^{}\left(x_{k-1}\right)+w_{k-1},$$

(64) $$z_{k}=h_{k}^{}\left(x_{k}\right)+v_{k},$$

where *f*_k_ and *h_k_* are known nonlinear functions, *w_k_*_−1_ and *v_k_* are additional noises, which follow zero-mean Student’s t distribution with scale matrix *Q_k_*_−1_ and *R_k_*, respectively.

Different from the GM-PHD filter to cope with nonlinear problems via giving the numerical solution to Gaussian integrals, the core problem of the STM-PHD filter for nonlinear systems is how to compute the Student’s t integrals. Some researchers have given numerical solutions to Student’s t integrals based on Taylor linearization, unscented transform or cubature rule [19,21,22]. Compared with Taylor linearization and unscented transform, the cubature rule-based filter is a derivative-free and vigorous method [25], so this paper utilizes the cubature rule to extend the STM-PHD filter to nonlinear system according to [22]. The proposed algorithm for handle nonlinearity is given as Table A2 in Appendix A.

## 4. Simulations and Results

To illustrate the performance of the proposed filter, simulation examples are designed to compare with standard GM-PHD filter in linear and nonlinear scenarios, respectively. To compare the performance of two filters, we choose the Optimal Sub-pattern Assignment (OSPA) distance as the metric, which can comprehensively measure the cardinality and localization errors [26]. The OSPA distance is defined as follows. Let $d^{\left(c\right)}(x,y):=\;\min(c,\left\|x-y\right\|)$ for x,y ∈ W, and $\Pi_{k}$ denotes the set of permutations on {1,2,…,k} for any k∈ℕ={1,2,…}. For p≥1, c>0, and arbitrary finite subsets $X=\{x_{1},\ldots ,x_{m}\}$ and $Y=\{y_{1},\ldots ,y_{n}\}$ belong to W, where $m,n\in {\mathbb{N}}_{0}=\{0,1,2,\ldots \}$:

(65) $${\bar{d}}_{p}^{\left(c\right)}(X,Y):=\;{\left(\frac{1}{n}\left(\underset{\pi \in \Pi_{n}}{\min}\sum_{i=1}^{m}d^{\left(c\right)}{\left(x_{i},y_{\pi \left(i\right)}\right)}^{p}+c^{p}\left(n-m\right)\right)\right)}^{\frac{1}{p}},$$

If m<n, and ${\bar{d}}_{p}^{\left(c\right)}(X,Y):={\bar{d}}_{p}^{\left(c\right)}(Y,X)$ if m>n; and ${\bar{d}}_{p}^{\left(c\right)}(X,Y)={\bar{d}}_{p}^{\left(c\right)}(Y,X)=0$ if m=n=0. p is the order parameter that determines the sensitivity to outliers and c is the cut-off parameter that determines the relative weighting of the penalties assigned to cardinality and localization errors. The details to choose the parameters p and c can be seen in [26]. In our simulation examples, we set p=2 and c=100.

### 4.1. Linear Scenario

Consider a two-dimensional scenario where there are twelve targets over region [−1000,1000] × [−1000,1000] during the interval of 100 s. Assuming no target spawning and each target moves as a constant velocity model similar to [9] with:

F=[1T000100001T0001], Q=[T34T3200T32T0000T34T3200T32T]q2,

where T=1 and q=5. The state $x_{k}={\left[p_{x,k},{p^{\prime }}_{x,k},p_{y,k},{p^{\prime }}_{y,k}\right]}^{T}$ of each target consist of position $\left[p_{x,k},p_{y,k}\right]$ and velocity $\left[{p^{\prime }}_{x,k},{p^{\prime }}_{y,k}\right]$ at time *k*. Their corresponding initial state and life time of each target are given as Table 1.

The noisy measurement model is the same as [9] with:

$$H=\left[\begin{array}{c} \begin{array}{cccc} 1 & 0 & 0 & 0 \end{array}\\ \begin{array}{cccc} 0 & 0 & 1 & 0 \end{array} \end{array}\right],\;R=\left[\begin{array}{cc} 100 & 0\\ 0 & 100 \end{array}\right],$$

The process and measurement noises with heavy tails are given as (66) and (67):

(66) $$w_{k}∼\{\begin{array}{l} N(0,Q)\;\mathrm{with}\;\mathrm{probability}\;0.99\\ N(0,25Q)\;\mathrm{with}\;\mathrm{probability}\;0.01 \end{array},$$

(67) $$v_{k}∼\{\begin{array}{l} N(0,R)\;\mathrm{with}\;\mathrm{probability}\;0.99\\ N(0,25R)\;\mathrm{with}\;\mathrm{probability}\;0.01 \end{array},$$

For the process and measurement noises in (66) and (67), about one percent of process and measurement noise values are drawn from Gaussian with severely high covariance. This percentage is also called contaminated rate which can be denoted by ε [27].

**Figure 2:**
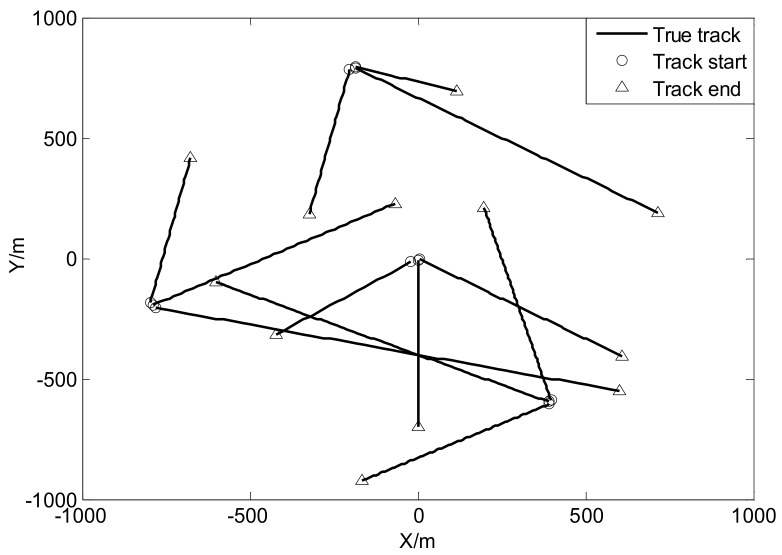
True trajectories of each target.

**Figure 3:**
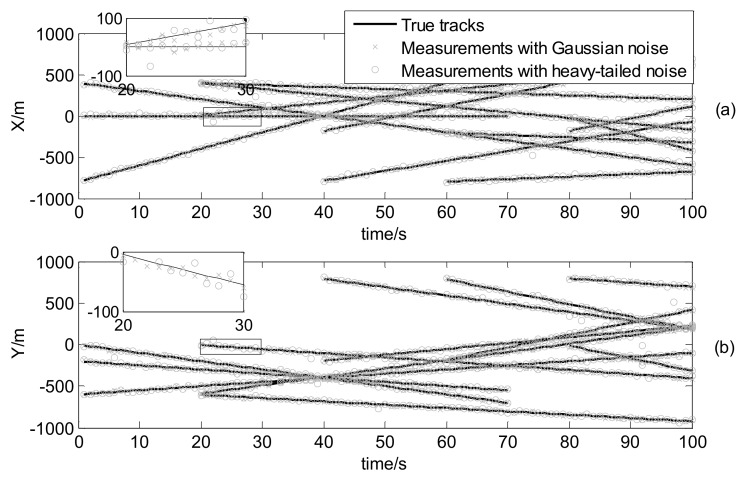
Measurements and true target positions versus time: (**a**) in x coordinate; (**b**) in y coordinate.

Assuming no spawned target and birth targets appear spontaneously according to a Poisson point process with intensity function:

(68) $$\gamma_{k}(x)=0.03\sum_{i=1}^{4}St(x;m_{\gamma }^{\left(i\right)},P_{\gamma }^{},\upsilon_{3}),$$

where $m_{\gamma }^{\left(1\right)}={\left[0,\;0,\;0,\;0\right]}^{T}$, $m_{\gamma }^{\left(2\right)}={\left[400,0,-600,0\right]}^{T}$, $m_{\gamma }^{\left(3\right)}={\left[-800,0,-200,0\right]}^{T}$ and $m_{\gamma }^{\left(4\right)}={\left[-200,0,800,0\right]}^{T}$, $\frac{\upsilon_{3}}{\upsilon_{3}-2}P_{\gamma }=\mathrm{diag}({\left[100,100,100,100\right]}^{T})$ and $\upsilon_{3}=10$. The true trajectories of each target are shown in Figure 2, while Figure 3 plots these trajectories with Gaussian measurements and heavy-tailed measurements over time (not plot clutter in figure). From Figure 3, it is can be seen that the individual heavy-tailed measurements obviously bias the true position compared with the corresponding Gaussian measurements, which may degrade the estimation accuracy.

The detection probability and target survival probability are $p_{D,k}^{}=0.98$ and $p_{S,k}^{}=0.99$, respectively. Truncated threshold, merged threshold and the maximum Student’s t components related to pruning and merging process are $T_{p}^{}=10^{-5}$, U=4 and $J_{\max}=100$, respectively. For simplification, set $\upsilon_{1}=\upsilon_{2}=\upsilon_{3}=10$.

**Figure 4:**
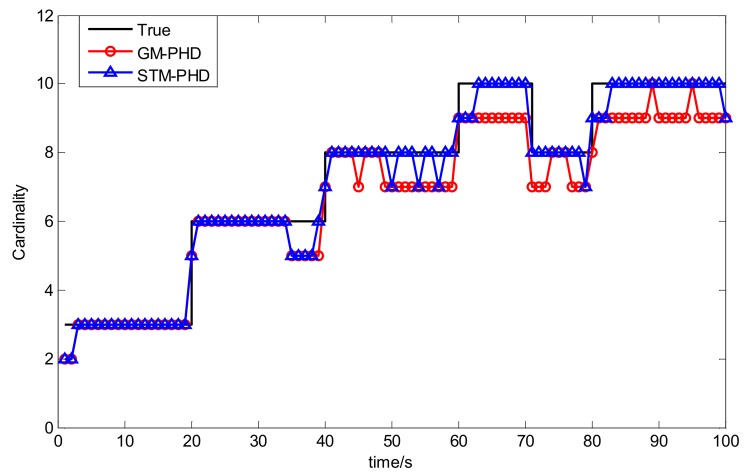
Comparison of cardinality estimation of two filters with fixed clutter rate (λ_c_ = 20).

**Figure 5:**
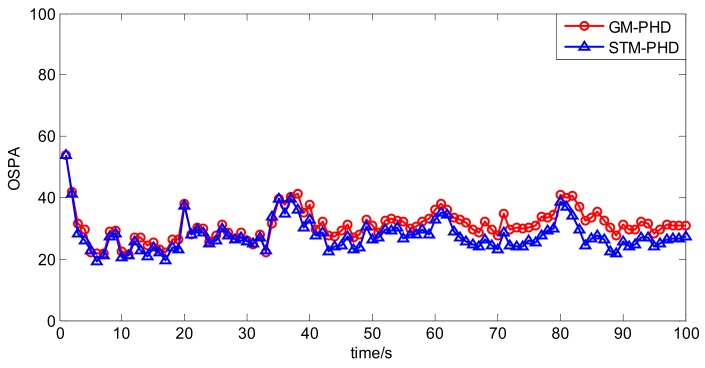
Comparison of OSPA distance of two filters with fixed clutter rate (λ_c_ = 20).

To evaluate the performance of the STM-PHD filter, we compare it with GM-PHD filter over 100 Monte Carlo (MC) trails with fixed clutter density. Under the uniform distribution assumption, the clutter density can be given by clutter rate λ_c_ with the relationship $\kappa_{k}(z)$ = λ_c_/*V*. In this simulation, we set λ_c_ = 20 (giving an average of 20 clutter returns per scan). Figure 4 and Figure 5 respectively show the estimated cardinality and the OSPA distance for two filters. The result in Figure 4 shows that the STM-PHD filter provides a noticeable improvement in terms of cardinality estimation accuracy compared with the GM-PHD filter, although some biased cardinality estimates appear for the STM-PHD filter.

In Figure 5 the OSPA distance of the STM-PHD filter is lower than that of the GM-PHD filter. Especially after 40 s more targets appear, difference of the OSPA distance between two filters is more noticeable. The main reason is that the STM-PHD filter has more accurate cardinality estimation.

**Figure 6:**
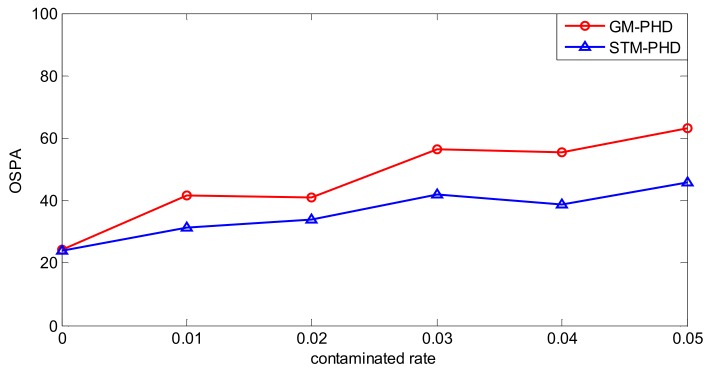
Comparison of OSPA distance of two filters with different contaminated rate.

To evaluate the performance of the proposed filter sufficiently, a simulation is executed over 100 MC trials with different contamination rates from ε = 0 to ε = 0.05. Then the time averaged OSPA distance of the STM-PHD filter and the GM-PHD filter, respectively, are shown in Figure 6.

From Figure 6, it can be seen that the time averaged OSPA distance of the STM-PHD filter is lower than that of the GM-PHD filter overall. The time averaged OSPA distances of two filters increases with the increasing contamination rate. Remarkably, the gap of OSPA distance between the STM-PHD filter and the GM-PHD filter changes wider from ε = 0 to ε = 0.05. It means that the STM-PHD filter has strong robustness against the negative effect of outliers, especially for high contamination rates. This is due to the fact the Student’s t noise model in the proposed approach can match the heavy-tailed non-Gaussian noise well. On the contrary, the Gaussian-based GM approach matches such a non-Gaussian noise worse and worse with the increasing of contaminated rate. Additionally, at ε = 0, the OSPA distance for the STM-PHD filter is the same as the GM-PHD filter. It indicates that the STM-PHD filter and the GM-PHD filter have the same tracking performance when outliers do not exist.

**Figure 7:**
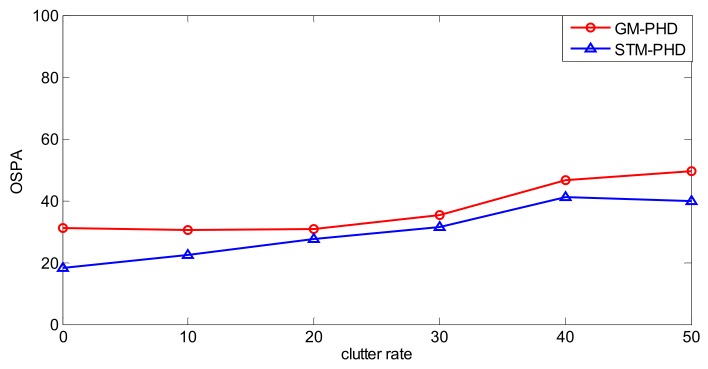
Comparison of OSPA distance for two filters with different clutter rate.

To further evaluate the performance of the proposed filter, a simulation is performed over 100 MC trails with different clutter rates from λ_c_ = 0 to λ_c_ = 50. The resulting time averaged OSPA distances of the proposed filter and the GM-PHD filter are shown in Figure 7. It can be seen that the time averaged OSPA distances of two filters increase with the increasing clutter rate and the time averaged OSPA distance of the STM-PHD filter is always lower than that of the GM-PHD filter under different clutter rates. This means that the STM-PHD filter generally outperforms the GM-PHD filter when outliers exist, no matter what the clutter rate is.

In addition, the computational cost for the STM-PHD filter lies at the same level as that of the GM-PHD filter for the linear system. Running on a computer with an Intel(R) Core(TM) i5-4570 CPU at 3.2 GHz, the average computing times per execution of the GM-PHD filter and the STM-PHD filter with different clutter rate are given in Table 2.

### 4.2. Nonlinear Scenario

In this example, we assume a maximum of ten targets appears on the observation region [−π,π] × [0,2000] and a nearly constant turn state model and nonlinear bearings and range measurement model are considered according to [9]. The state ${\tilde{x}}_{k}={\left[x_{k}^{T},\omega_{k}\right]}^{T}$ consists of position and velocity $x_{k}={\left[p_{x,k},{p^{\prime }}_{x,k},p_{y,k},{p^{\prime }}_{y,k}\right]}^{T}$ as well as the turn rate $\omega_{k}$. The state model is given by:

(69) $$\begin{array}{l} x_{k}=F(\omega_{k-1})x_{k-1}+Gw_{k-1}\\ \omega_{k}=\omega_{k-1}+Tu_{k-1} \end{array},$$

where:

F(ω)=[1sinωT/ω0−(1−cosωT)ω0cosωT0−sinωT0(1−cosωT)ω1sinωT/ω0sinωT0cosωT], G=[T22T0000T22T],T=1,

The noisy measurement model with range and bearing measurement $z_{k}=[r_{k},\theta_{k}]$ is given by:

(70) $$z_{k}=\left[\begin{array}{c} \sqrt{p_{x,k}{}^{2}+p_{y,k}{}^{2}}\\ \arctan\frac{p_{y,k}}{p_{x,k}} \end{array}\right]+v_{k},$$

Like the linear scenario, the outliers contaminated process and measurement noises can be given by:

(71) $$w_{k}∼\{\begin{array}{l} N(0,Q)\;\mathrm{with}\;\mathrm{probability}\;0.97\\ N(0,25Q)\;\mathrm{with}\;\mathrm{probability}\;0.03 \end{array},$$

(72) $$v_{k}∼\{\begin{array}{l} N(0,R)\;\mathrm{with}\;\mathrm{probability}\;0.97\\ N(0,25R)\;\mathrm{with}\;\mathrm{probability}\;0.03 \end{array},$$

with $Q=\mathrm{diag}([5,5,\pi {/180]}^{T}{)}^{2}$, $R=\mathrm{diag}([10,2(\pi {/180)]}^{T}{)}^{2}$.

In the simulation, we assume no spawned target and that the birth target is Poisson with intensity:

(73) $$\gamma_{k}(x)=0.02\sum_{i=1}^{2}St(x;m_{\gamma }^{\left(i\right)},P_{\gamma }^{},\upsilon_{3})+0.03\sum_{i=3}^{4}St(x;m_{\gamma }^{\left(i\right)},P_{\gamma }^{},\upsilon_{3}),$$

where $m_{\gamma }^{\left(1\right)}={\left[-1500,0,250,0,0\right]}^{T}$, $m_{\gamma }^{\left(2\right)}={\left[-250,0,1000,0,0\right]}^{T}$, $m_{\gamma }^{\left(3\right)}={\left[250,0,750,0,0\right]}^{T}$ and $m_{\gamma }^{\left(4\right)}={\left[1000,0,1500,0,0\right]}^{T}$, $\frac{\upsilon_{3}}{\upsilon_{3}-2}P_{\gamma }=\mathrm{diag}{\left({\left[50,50,50,50,6(\pi /180)\right]}^{T}\right)}^{2}$ and $\upsilon_{3}=6$. (The unit of distance, angle and time in this paper are meter, radian and second, respectively.)

**Figure 8:**
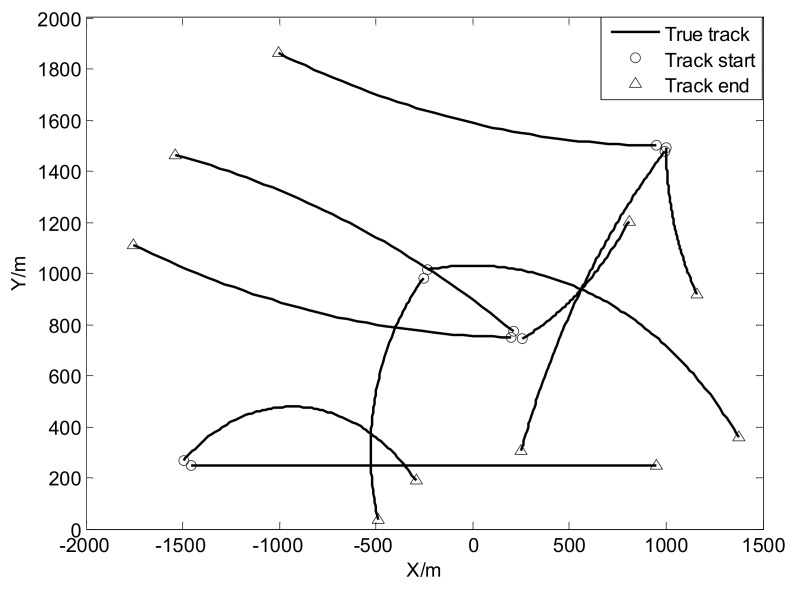
True trajectories of each target.

**Figure 9:**
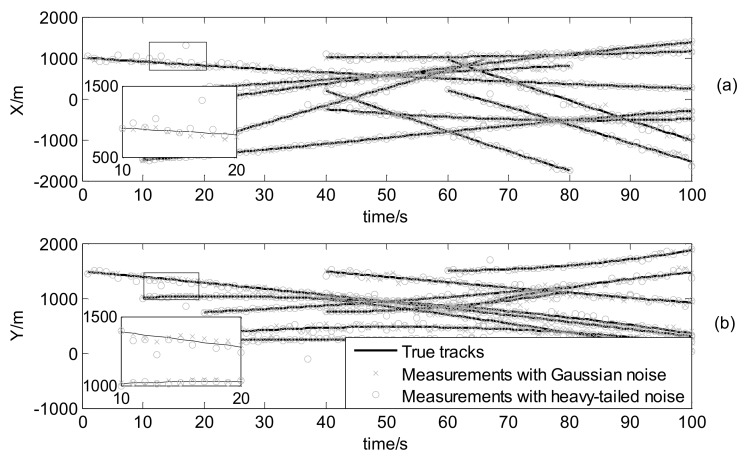
Measurements and true target positions versus time: (**a**) in x coordinate; (**b**) in y coordinate.

The initial target states are given by Table 3 and the true trajectories of each target are shown as Figure 8. In addition, Figure 9 plots corresponding measurements with Gaussian noise and heavy-tailed noise respectively over time (not plot clutter in figure). In Figure 9, it also shows the results analogous to the linear case as shown in Figure 3.

**Figure 10:**
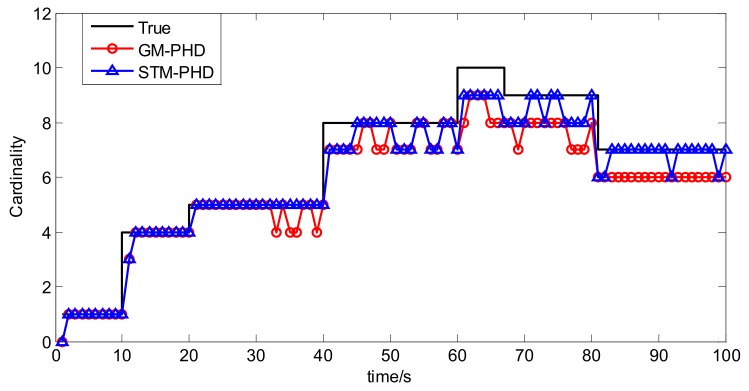
Comparison of cardinality estimation of two filters with fixed clutter rate (λ_c_ = 20).

**Figure 11:**
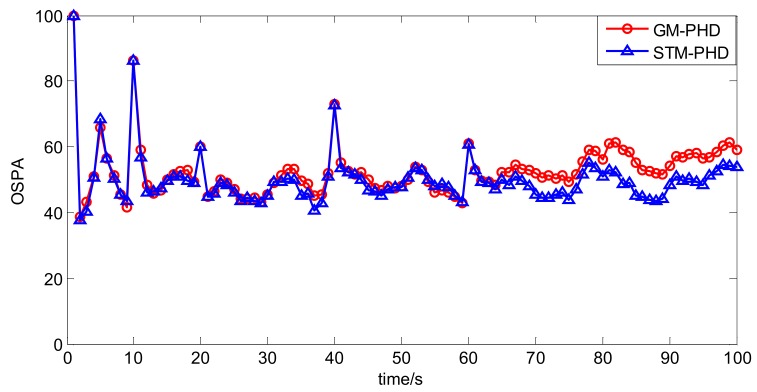
Comparison of OSPA distance of two filters with fixed clutter rate (λ_c_ = 20).

To evaluate the performance of the CKF based STM-PHD filter to cope with nonlinear problem, we compare it with CKF based GM-PHD filter [11] over 100 Monte Carlo (MC) trails with fixed clutter rate λ_c_ = 20. Figure 10 and Figure 11 respectively show the estimated cardinality and the OSPA distance of two filters. From Figure 10, it can be seen that the STM-PHD filter is superior to the GM-PHD filter in terms of cardinality estimation accuracy, although the STM-PHD filter has cardinality bias when the number of targets increases. The main reason for generating cardinality bias is that some necessary approximations for coping with nonlinear problems induce errors.

In Figure 11 the OSPA distances of the STM-PHD filter and the GM-PHD filter are at the same level before 65 s and later on the OSPA distance of the STM-PHD filter is obviously lower than that of the GM-PHD filter. This result matches the result in Figure 10 well. It indicates that the difference of OSPA distance between two filters is due to the difference of cardinality estimation accuracy.

**Figure 12:**
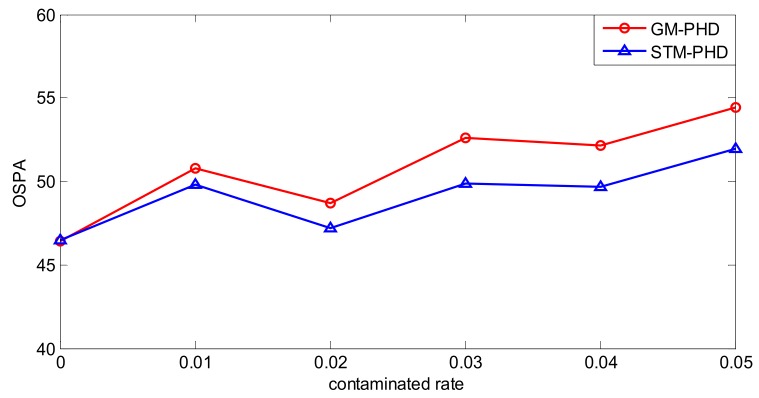
Comparison of OSPA distance of two filters with different contamination rates.

To evaluate the performance of the STM-PHD filter sufficiently, simulation is performed over 100 MC trials with different contaminated rate from ε = 0 to ε = 0.05. Then the time averaged OSPA distances of the STM-PHD filter and the GM-PHD filter are shown in Figure 12.

Analogous to the linear scenario, it can be seen that the time averaged OSPA distance of the STM-PHD filter is lower than that of the GM-PHD filter at almost all contaminated rates. The gap of the time averaged OSPA distance between the STM-PHD filter and the GM-PHD filter also changes widely as the contamination rates increase. Nevertheless, the gap is not noticeable like in the linear scenario. This is due to the relatively big approximation error induced in the nonlinear scenario. The result indicates that the STM-PHD filter still outperforms the GM-PHD filter to cope with outliers for nonlinear systems.

**Figure 13:**
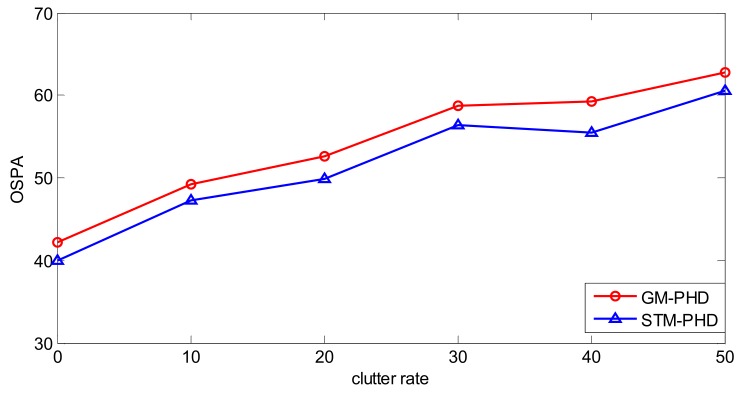
Comparison of OSPA distance of two filters with different clutter rates.

To further evaluate the performance of the proposed filter, 100 MC trails are performed from λ_c_ = 0 to λ_c_ = 50. The time averaged OSPA distances versus varying clutter rate for the STM-PHD filter and the GM-PHD filter are shown in Figure 13. It can be seen that the time averaged OSPA distance of the STM-PHD filter is lower than that of the GM-PHD filter at different clutter rates, and the trend for two filters goes up with the increase of clutter rate. Generally speaking, the STM-PHD filter is superior to the GM-PHD filter but the superiority is not noticeable like in the linear scenario. This is due to the fact the approximation error induced in a nonlinear scenario is bigger than that in a linear scenario. Nevertheless, the results still indicate that the STM-PHD filter is valid to handle the outliers.

Again, the computing time for each filter under the different clutter rate is given in Table 4. It shows that the STM-PHD filter has a higher computing cost compared to the GM-PHD filter and the higher the clutter rate is, much more time is consumed for the STM-PHD filter. The reason is that computing nonlinear Student’s t integrals is more complex than computing nonlinear Gaussian integrals.

## 5. Conclusions

To solve the problem that the process and measurement outliers degrade the performance of PHD filter, this paper proposes a Student’s t mixture-based PHD filter, where the prior multi-target intensities are approximated as a mixture of the Student’s t components to be propagated in time and the Student’s t mixture approximated posterior multi-target intensity are obtained through a Student’s t approximation-based recursion. The proposed approach can efficiently suppress the negative impact caused by the process and measurement outliers, while still maintaining good estimation accuracy. The advantages of the proposed filter are verified sufficiently by simulation. The simulation results also imply that the proposed approach outperforms the GM-based approach in scenarios where outliers appear due to electromagnetic interference or sensors’ own unreliability. In addition, the proposed approach is also suitable for the CPHD filter. However, the proposed approach has relatively high computational cost for nonlinear systems. In further study, we will try to improve this. Moreover, how to combine the proposed Student’s t mixture implementation with the multi-Bernoulli filters (including CBMeMBer filter and labeled RFS based multi-Bernoulli filter) is also our focus in the next work.

## Figures and Tables

**Table 1:** A list of initial target states.

Target Index	Life Time (s)	Initial States (m, m/s, m, m/s)
#1	(1, 70)	[0, 0, 0, −10]
#2	(1, 100)	[400, −10, −600, 5]
#3	(1, 70)	[−800, 20, −200, −5]
#4	(20, 100)	[400, −7, −600, −4]
#5	(20, 100)	[400, −2.5, −600, 10]
#6	(20, 100)	[0, 7.5, 0, −5]
#7	(40, 100)	[−800, 12, −200, 7]
#8	(40, 100)	[−200, −3, 800, −10]
#9	(60, 100)	[−800, 3, −200, 15]
#10	(60, 100)	[−200, −3, 800, −15]
#11	(80, 100)	[0, −20, 0, −15]
#12	(80, 100)	[−200, 15, 800, −5]

**Table 2:** Average computing time with different clutter rates.

	Clutter Rate					
	0	10	20	30	40	50
GM-PHD	0.9217 s	0.9917 s	1.0782 s	1.1253 s	1.1938 s	1.2068 s
STM-PHD	0.9146 s	0.9961 s	1.0780 s	1.1417 s	1.2576 s	1.2486 s

**Table 3:** A list of initial target states.

Target Index	Life Time (s)	Initial States (m, m/s, m, m/s, rad/s)
#1	(1, 100)	[1000, −10, 1500, −10, 2π/(180 × 8)]
#2	(10, 100)	[−250, 20, 1000, 3, −2π/(180 × 3)]
#3	(10, 100)	[−1500, 11, 250, 10, −2π/(180 × 2)]
#4	(10, 66)	[−1500, 43, 250, 0, 0]
#5	(20, 80)	[250, 11, 750, 5, 2π/(180 × 4)]
#6	(40, 100)	[−250, −12, 1000, −12, 2π/(180 × 2)]
#7	(40, 100)	[1000, 0, 1500, −10, 2π/(180 × 4)]
#8	(40, 80)	[250, −50, 750, 0, −2π/(180 × 4)]
#9	(60, 100)	[1000, −50, 1500, 00, −2π/180 × 4]
#10	(60, 100)	[250, −40, 750, 25, 2π/(180 × 4)]

**Table 4:** Average computing time with different clutter rate.

	Clutter Rate					
	0	10	20	30	40	50
GM-PHD	1.0142 s	1.3780 s	1.8481 s	2.4988 s	3.3321 s	3.9051 s
STM-PHD	1.8534 s	3.3179 s	5.4081 s	8.5213 s	12.9163 s	16.6113 s

## Appendix A

**Table A1:** Pseudocode for the STM-PHD filter.

$\begin{array}{l} \mathrm{given}\;{\left\{w_{k-1}^{\left(i\right)},m_{k-1}^{\left(i\right)},P_{k-1}^{\left(i\right)}\right\}}_{i=1}^{J_{k-1}},\;\mathrm{the}\;\mathrm{measurement}\;\mathrm{set}\;Z_{k}\;\mathrm{and}\;\mathrm{the}\;\mathrm{degree}\;\mathrm{of}\;\mathrm{freedom}\;\mathrm{parameters}\;\upsilon_{1},\upsilon_{2},\upsilon_{3}\;.\\ \mathrm{step}1.(\mathrm{prediction}\;\mathrm{for}\;\mathrm{birth}\;\mathrm{targets})\\ \;i=0.\\ \;\mathrm{for}\;j=1,\ldots ,J_{\gamma ,k}\\ \;i:=i+1.\\ \;w_{k|k-1}^{\left(i\right)}=w_{\gamma ,k}^{\left(j\right)},\;m_{k|k-1}^{\left(i\right)}=m_{\gamma ,k}^{\left(j\right)},\;P_{k|k-1}^{\left(i\right)}=P_{\gamma ,k}^{\left(j\right)},\\ \;\mathrm{end}\\ \;\mathrm{for}\;j=1,\ldots ,J_{\beta ,k}\\ \;\mathrm{for}\;l=1,\ldots ,J_{k-1}\\ \;i:=i+1.\\ \;w_{k|k-1}^{\left(i\right)}=w_{k-1}^{\left(l\right)}w_{\beta ,k}^{\left(j\right)},\;m_{k|k-1}^{\left(i\right)}=F_{\beta ,k-1}^{\left(j\right)}m_{k-1}^{\left(l\right)}+d_{\beta ,k-1}^{\left(j\right)},\\ \;P_{k|k-1}^{\left(i\right)}=F_{\beta ,k-1}^{\left(j\right)}P_{k-1}^{\left(l\right)}{\left(F_{\beta ,k-1}^{\left(j\right)}\right)}^{T}+\frac{\upsilon_{1}(\upsilon_{3}-2)}{\upsilon_{3}(\upsilon_{1}-2)}Q_{\beta ,k-1}^{},\\ \;\mathrm{end}\\ \;\mathrm{end}\\ \mathrm{step}2.\;(\mathrm{prediction}\;\mathrm{for}\;\mathrm{existing}\;\mathrm{targets})\\ \;\mathrm{for}\;j=1,\ldots ,J_{k-1}\\ \;i:=i+1.\\ \;w_{k|k-1}^{\left(i\right)}=p_{S,k}w_{k-1}^{\left(j\right)},\\ \;m_{k|k-1}^{\left(i\right)}=F_{k-1}^{\left(j\right)}m_{k-1}^{\left(j\right)},\;P_{k|k-1}^{\left(i\right)}=F_{k-1}^{}P_{k-1}^{\left(j\right)}F_{k-1}^{T}+\frac{\upsilon_{1}(\upsilon_{3}-2)}{\upsilon_{3}(\upsilon_{1}-2)}Q_{k-1}^{},\\ \;\mathrm{end}\\ \;J_{k|k-1}=i.\\ \mathrm{step}3.(\mathrm{construction}\;\mathrm{of}\;\mathrm{PHD}\;\mathrm{update}\;\mathrm{components})\\ \;\mathrm{for}\;j=1,\ldots ,J_{k|k-1}\\ \;\eta_{k|k-1}^{\left(j\right)}=H_{k}m_{k|k-1}^{\left(j\right)},\;P_{zz,k|k-1}^{\left(j\right)}=H_{k}^{}P_{k|k-1}^{\left(j\right)}H_{k}^{T}+\frac{\upsilon_{2}(\upsilon_{3}-2)}{\upsilon_{3}(\upsilon_{2}-2)}R_{k}^{},\\ \;K_{k}^{\left(j\right)}=P_{k|k-1}^{\left(j\right)}H_{k}^{T}{\left[P_{zz,k|k-1}^{\left(j\right)}\right]}^{-1},\;P_{k|k}^{\left(j\right)}=P_{k|k-1}^{\left(j\right)}-K_{k}^{\left(j\right)}H_{k}^{}P_{k|k-1}^{\left(j\right)},\\ \;\mathrm{end}\\ \mathrm{step}4.(\mathrm{update})\\ \;\mathrm{for}\;j=1,\ldots ,J_{k|k-1}\\ \;w_{k}^{\left(j\right)}=(1-p_{D,k})w_{k|k-1}^{\left(j\right)},\\ \;m_{k}^{\left(j\right)}=m_{k|k-1}^{\left(j\right)},\;P_{k}^{\left(j\right)}=P_{k|k-1}^{\left(j\right)},\\ \;\mathrm{end}\\ \;l=0.\\ \;\mathrm{for}\;\mathrm{each}\;z\in Z_{k}\\ \;l:=l+1.\\ \;\mathrm{for}\;j=1,\ldots ,J_{k|k-1}\\ \;\Delta^{\left(lJ_{k|k-1}+j\right)}=\sqrt{{\left(z-\eta_{k|k-1}^{\left(j\right)}\right)}^{T}{\left(P_{zz,k|k-1}^{\left(j\right)}\right)}^{-1}(z-\eta_{k|k-1}^{\left(j\right)})}\\ \;w_{k}^{\left(lJ_{k|k-1}+j\right)}=p_{D,k}w_{k|k-1}^{\left(j\right)}St(z;\eta_{k|k-1}^{\left(j\right)},S_{k}^{\left(j\right)},\upsilon_{3}),\\ \;{m^{\prime }}_{k}^{\left(lJ_{k|k-1}+j\right)}=m_{k|k-1}^{\left(j\right)}+K_{k}^{\left(j\right)}(z-\eta_{k|k-1}^{\left(j\right)}),\\ \;{P^{\prime }}_{k}^{\left(lJ_{k|k-1}+j\right)}=\frac{\upsilon_{3}+{\left(\Delta^{\left(lJ_{k|k-1}+j\right)}\right)}^{2}}{\upsilon_{3}+d_{z}}P_{k|k}^{\left(j\right)},\\ \;\mathrm{end}\\ \;w_{k}^{\left(lJ_{k|k-1}+j\right)}:=\frac{w_{k}^{\left(lJ_{k|k-1}+j\right)}}{\kappa_{k}(z)+\sum_{i=1}^{J_{k|k-1}}w_{k}^{\left(lJ_{k|k-1}+i\right)}},\;\mathrm{for}\;j=1,\ldots ,J_{k|k-1}.\\ \;\mathrm{end}\\ \;{\upsilon^{\prime }}_{3}=\upsilon_{3}+d_{z},\;J_{k}=lJ_{k|k-1}+J_{k|k-1}.\\ \mathrm{step}5.(\mathrm{moment}\;\mathrm{matching})\\ \;\mathrm{for}\;j=1,\ldots ,J_{k}\\ \;m_{k}^{\left(j\right)}={m^{\prime }}_{k}^{\left(j\right)},\;P_{k}^{\left(j\right)}=\frac{{\upsilon^{\prime }}_{3}(\upsilon_{3}-2)}{\upsilon_{3}({\upsilon^{\prime }}_{3}-2)}{P^{\prime }}_{k}^{\left(j\right)},\\ \;\mathrm{end}\\ \mathrm{output}{\left\{w_{k}^{\left(i\right)},m_{k}^{\left(i\right)},P_{k}^{\left(i\right)}\right\}}_{i=1}^{J_{k}} \end{array}$

**Table A2:** Pseudocode for the cubature rule based STM-PHD filter.

$\begin{array}{l} \mathrm{given}\;{\left\{w_{k-1}^{\left(i\right)},m_{k-1}^{\left(i\right)},P_{k-1}^{\left(i\right)}\right\}}_{i=1}^{J_{k-1}},\;\mathrm{the}\;\mathrm{measurement}\;\mathrm{set}\;Z_{k}\;\mathrm{and}\;\mathrm{the}\;\mathrm{degree}\;\mathrm{of}\;\mathrm{freedom}\;\mathrm{parameters}\;\upsilon_{1},\upsilon_{2},\upsilon_{3}\;.\\ \mathrm{step}1.(\mathrm{prediction}\;\mathrm{for}\;\mathrm{birth}\;\mathrm{targets})\\ \;\mathrm{follow}\;\mathrm{Step}\;1.\;\mathrm{of}\;\mathrm{table}.\\ \;\mathrm{for}\;j=1,\ldots ,i \end{array}$
$\begin{array}{l} \;-\;\mathrm{use}\;\mathrm{the}\;\mathrm{cubature}\;\mathrm{rule}\;\mathrm{suitable}\;\mathrm{for}\text{ Student′s }t\;\mathrm{integral} \end{array}$ (see [22]) $\begin{array}{l} \mathrm{with}\;m_{k-1}^{\left(j\right)}\;\mathrm{and}\;P_{k-1}^{\left(j\right)}\; \end{array}$
$\begin{array}{l} \;\mathrm{to}\;\mathrm{obtain}\;a\;\mathrm{set}\;\mathrm{of}\;\mathrm{cubature}\;\mathrm{points}\;\mathrm{and}\;\mathrm{weights},\;\mathrm{denoted}\;\mathrm{by}\;{\left\{x_{k-1}^{\left(l\right)},\mu_{}^{\left(l\right)}\right\}}_{l=1}^{L}\\ \;-\;\mathrm{compute}\\ \;x_{k|k-1}^{\left(l\right)}:=f_{\beta ,k}(x_{k-1}^{\left(l\right)}),\;l=1,\ldots ,L,\\ \;m_{k|k-1}^{\left(j\right)}=\sum_{l=1}^{L}\mu_{}^{\left(l\right)}x_{k|k-1}^{\left(l\right)},\\ \;P_{k|k-1}^{\left(j\right)}=\frac{\upsilon_{3}-2}{\upsilon_{3}}\sum_{l=1}^{L}\mu_{k-1}^{\left(l\right)}(x_{k|k-1}^{\left(l\right)}-m_{k|k-1}^{\left(j\right)}){\left(x_{k|k-1}^{\left(l\right)}-m_{k|k-1}^{\left(j\right)}\right)}^{T}+\frac{\upsilon_{1}(\upsilon_{3}-2)}{\upsilon_{3}(\upsilon_{1}-2)}Q_{\beta ,k-1}^{},\\ \;\mathrm{end}\\ \mathrm{step}2.\;(\mathrm{prediction}\;\mathrm{for}\;\mathrm{existing}\;\mathrm{targets})\\ \;\mathrm{for}\;j=1,\ldots ,J_{k-1}\\ \;i:=i+1.\\ \;w_{k|k-1}^{\left(i\right)}=p_{S,k}w_{k-1}^{\left(j\right)},\\ \;-\;\mathrm{use}\;\mathrm{the}\;\mathrm{cubature}\;\mathrm{rule}\;\mathrm{suitable}\;\mathrm{for}\text{ Student′s }t\;\mathrm{integral}\;\mathrm{with}\;m_{k-1}^{\left(j\right)}\;\mathrm{and}\;P_{k-1}^{\left(j\right)}\;\\ \;\mathrm{to}\;\mathrm{obtain}\;a\;\mathrm{set}\;\mathrm{of}\;\mathrm{cubature}\;\mathrm{points}\;\mathrm{and}\;\mathrm{weights},\;\mathrm{denoted}\;\mathrm{by}\;{\left\{x_{k-1}^{\left(l\right)},\mu_{}^{\left(l\right)}\right\}}_{l=1}^{L}\\ \;-\;\mathrm{compute}\\ \;x_{k|k-1}^{\left(l\right)}:=f_{k}(x_{k-1}^{\left(l\right)}),\;l=1,\ldots ,L,\\ \;m_{k|k-1}^{\left(j\right)}=\sum_{l=1}^{L}\mu_{k-1}^{\left(l\right)}x_{k|k-1}^{\left(l\right)},\\ \;P_{k|k-1}^{\left(j\right)}=\frac{\upsilon_{3}-2}{\upsilon_{3}}\sum_{l=1}^{L}\mu^{\left(l\right)}(x_{k|k-1}^{\left(l\right)}-m_{k|k-1}^{\left(j\right)}){\left(x_{k|k-1}^{\left(l\right)}-m_{k|k-1}^{\left(j\right)}\right)}^{T}+\frac{\upsilon_{1}(\upsilon_{3}-2)}{\upsilon_{3}(\upsilon_{1}-2)}Q_{k-1}^{},\\ \;\mathrm{end}\\ \;J_{k|k-1}=i.\\ \mathrm{step}3.(\mathrm{construction}\;\mathrm{of}\;\mathrm{PHD}\;\mathrm{update}\;\mathrm{components})\\ \;\mathrm{for}\;j=1,\ldots ,J_{k|k-1}\\ \;-\;\mathrm{compute}\\ \;z_{k|k-1}^{\left(l\right)}:=h_{k}(x_{k|k-1}^{\left(l\right)}),\;l=1,\ldots ,L,\\ \;\eta_{k|k-1}^{\left(j\right)}=\sum_{l=1}^{L}\mu_{}^{\left(l\right)}z_{k|k-1}^{\left(l\right)},\\ \;P_{zz,k|k-1}^{\left(j\right)}=\frac{\upsilon_{3}-2}{\upsilon_{3}}\sum_{l=1}^{L}\mu^{\left(l\right)}(z_{k|k-1}^{\left(l\right)}-\eta_{k|k-1}^{\left(j\right)}){\left(z_{k|k-1}^{\left(l\right)}-\eta_{k|k-1}^{\left(j\right)}\right)}^{T}+\frac{\upsilon_{2}(\upsilon_{3}-2)}{\upsilon_{3}(\upsilon_{2}-2)}R_{k}^{},\\ \;P_{xz,k|k-1}^{\left(j\right)}=\frac{\upsilon_{3}-2}{\upsilon_{3}}\sum_{l=1}^{L}\mu^{\left(l\right)}(x_{k|k-1}^{\left(l\right)}-m_{k|k-1}^{\left(j\right)}){\left(z_{k|k-1}^{\left(l\right)}-\eta_{k|k-1}^{\left(j\right)}\right)}^{T}\\ \;K_{k}^{\left(j\right)}=P_{xz,k|k-1}^{\left(j\right)}{\left[P_{zz,k|k-1}^{\left(j\right)}\right]}^{-1},\\ \;P_{k|k}^{\left(j\right)}=P_{k|k-1}^{\left(j\right)}-P_{xz,k|k-1}^{\left(j\right)}{\left[P_{zz,k|k-1}^{\left(j\right)}\right]}^{-1}{\left[P_{xz,k|k-1}^{\left(j\right)}\right]}^{T},\\ \;\mathrm{end}\\ \mathrm{step}4.(\mathrm{update})\;\mathrm{and}\mathrm{step}5.(\mathrm{moment}\;\mathrm{matching})\\ \;\mathrm{follow}\;\mathrm{Step}\;4.\;\mathrm{and}\;\mathrm{Step}\;5.\;\mathrm{of}\;\mathrm{table}\;\mathrm{to}\;\mathrm{obtain}\;{\left\{w_{k}^{\left(i\right)},m_{k}^{\left(i\right)},P_{k}^{\left(i\right)}\right\}}_{i=1}^{J_{k}}.\\ \mathrm{output}\;{\left\{w_{k}^{\left(i\right)},m_{k}^{\left(i\right)},P_{k}^{\left(i\right)}\right\}}_{i=1}^{J_{k}}. \end{array}$

## Appendix B

Proof of Proposition 1:

$$\begin{array}{lll} D_{k|k-1}(x) & = & \int (p_{s,k}St(x;F_{k-1}^{}u,Q_{k-1}^{},\upsilon_{1})+\sum_{l=1}^{J_{\beta ,k}}w_{\beta ,k}^{\left(l\right)}St(x;F_{\beta ,k-1}^{\left(l\right)}u+d_{\beta ,k-1}^{\left(l\right)},Q_{\beta ,k-1}^{\left(l\right)},\upsilon_{3}))\sum_{j=1}^{J_{k-1}}w_{k-1}^{\left(j\right)}St(u;m_{k-1}^{\left(j\right)},P_{k-1}^{\left(j\right)},\upsilon_{3})du\\ & & +\sum_{i=1}^{J_{\gamma ,k}}w_{\gamma ,k}^{\left(i\right)}St(x;m_{\gamma ,k}^{\left(i\right)},P_{\gamma ,k}^{},\upsilon_{3})\\ & = & p_{s,k}\sum_{j=1}^{J_{k-1}}w_{k-1}^{\left(j\right)}\int St(x;F_{k-1}^{}u,Q_{k-1}^{},\upsilon_{1})St(u;m_{k-1}^{\left(j\right)},P_{k-1}^{\left(j\right)},\upsilon_{3})du\\ & & +\sum_{j=1}^{J_{k-1}}\sum_{l=1}^{J_{\beta ,k}}w_{k-1}^{\left(j\right)}w_{\beta ,k}^{\left(l\right)}\int St(x;F_{\beta ,k-1}^{\left(l\right)}u+d_{\beta ,k-1}^{\left(l\right)},Q_{\beta ,k-1}^{\left(l\right)},\upsilon_{3})St(u;m_{k-1}^{\left(j\right)},P_{k-1}^{\left(j\right)},\upsilon_{3})du\\ & & +\sum_{i=1}^{J_{\gamma ,k}}w_{\gamma ,k}^{\left(i\right)}St(x;m_{\gamma ,k}^{\left(i\right)},P_{\gamma ,k}^{},\upsilon_{3})\\ & = & p_{s,k}\sum_{j=1}^{J_{k-1}}w_{k-1}^{\left(j\right)}St(x;F_{k-1}m_{k-1}^{\left(j\right)},F_{k-1}P_{k-1}^{\left(j\right)}F_{k-1}^{T}+\frac{\upsilon_{3}-2}{\upsilon_{3}}\frac{\upsilon_{1}}{\upsilon_{1}-2}Q_{k-1},\upsilon_{3})\\ & & +\sum_{j=1}^{J_{k-1}}\sum_{l=1}^{J_{\beta ,k}}w_{k-1}^{\left(j\right)}w_{\beta ,k}^{\left(l\right)}St(x;F_{\beta ,k-1}^{\left(l\right)}m_{k-1}^{\left(j\right)}+d_{\beta ,k-1}^{\left(l\right)},F_{\beta ,k-1}^{\left(l\right)}P_{\beta ,k-1}^{\left(l\right)}{\left(F_{\beta ,k-1}^{\left(l\right)}\right)}^{T}+\frac{\upsilon_{3}-2}{\upsilon_{3}}\frac{\upsilon_{1}}{\upsilon_{1}-2}Q_{\beta ,k-1},\upsilon_{3})\\ & & +\sum_{i=1}^{J_{\gamma ,k}}w_{\gamma ,k}^{\left(i\right)}St(x;m_{\gamma ,k}^{\left(i\right)},P_{\gamma ,k}^{},\upsilon_{3}). \end{array}$$

Proof of Proposition 2:

$$\begin{array}{lll} D_{k}(x) & = & \left(1-p_{D,k})D_{k|k-1}(x\right)\\ & & +\sum_{z\in Z_{k}}\frac{p_{D,k}St(z;H_{k}^{}x,R_{k}^{},\upsilon_{2})\sum_{j=1}^{J_{k|k-1}}w_{k|k-1}^{\left(j\right)}St(x;m_{k|k-1}^{\left(j\right)},P_{k|k-1}^{\left(j\right)},\upsilon_{3})}{\kappa_{k}(z)+\int p_{D,k}St(z;H_{k}^{}x,R_{k}^{},\upsilon_{2})\sum_{l=1}^{J_{k|k-1}}w_{k|k-1}^{\left(l\right)}St(u;m_{k|k-1}^{\left(l\right)},P_{k|k-1}^{\left(l\right)},\upsilon_{3})du}\\ & = & \left(1-p_{D,k})D_{k|k-1}(x\right)\\ & & +\sum_{z\in Z_{k}}\frac{p_{D,k}\sum_{j=1}^{J_{k|k-1}}w_{k|k-1}^{\left(j\right)}St(z;H_{k}^{}x,R_{k}^{},\upsilon_{2})St(x;m_{k|k-1}^{\left(j\right)},P_{k|k-1}^{\left(j\right)},\upsilon_{3})}{\kappa_{k}(z)+p_{D,k}\sum_{l=1}^{J_{k|k-1}}w_{k|k-1}^{\left(j\right)}\int St(z;H_{k}^{}x,R_{k}^{},\upsilon_{2})St(u;m_{k|k-1}^{\left(l\right)},P_{k|k-1}^{\left(l\right)},\upsilon_{3})du}, \end{array}$$

Using Lamma 2:

$$\begin{array}{l} St(z;H_{k}^{}x,R_{k}^{},\upsilon_{2})St(x;m_{k|k-1}^{\left(j\right)},P_{k|k-1}^{\left(j\right)},\upsilon_{3})\\ =St\left(z;H_{k}^{}m_{k|k-1}^{\left(j\right)},H_{k}^{}P_{k|k-1}^{\left(j\right)}H_{k}^{T}+\frac{(\upsilon_{3}-2)\upsilon_{2}}{(\upsilon_{2}-2)\upsilon_{3}}R,\upsilon_{3}\right)\\ St\left(x;m_{k|k-1}^{\left(j\right)}+K_{k}^{\left(j\right)}(z-H_{k}m_{k|k-1}^{\left(j\right)}),\frac{\upsilon_{3}+\Delta^{2}}{\upsilon_{3}+d_{z}}(P_{k|k-1}^{\left(j\right)}-K_{k}^{\left(j\right)}H_{k}P_{k|k-1}^{\left(j\right)}),\upsilon_{3}+d_{z}\right)\\ =q_{k}^{\left(j\right)}\left(z\right)St(x;m_{k|k}^{\left(j\right)}\prime (z),P_{k|k}^{\left(j\right)}\prime (z),\upsilon_{3}\prime ), \end{array}$$

with:

$$\begin{array}{l} K_{k}^{\left(j\right)}=P_{k|k-1}^{\left(j\right)}H_{k}^{T}{\left(H_{k}P_{k|k-1}^{\left(j\right)}H_{k}^{T}+\frac{\upsilon_{3}-2}{\upsilon_{3}}\frac{\upsilon_{2}}{\upsilon_{2}-2}R_{k}\right)}^{-1},\\ \Delta^{2}={\left(z-H_{k}m_{k|k-1}^{\left(j\right)}\right)}^{T}{\left(P_{zz,k|k-1}^{\left(j\right)}\right)}^{-1}(z-H_{k}m_{k|k-1}^{\left(j\right)}), \end{array}$$

Using Lamma 1:

$$\int St(z;H_{k}^{}x,R_{k}^{},\upsilon_{2})St(u;m_{k|k-1}^{\left(l\right)},P_{k|k-1}^{\left(l\right)},\upsilon_{3})du=St\left(z;H_{k}^{}m_{k|k-1}^{\left(l\right)},H_{k}^{}P_{k|k-1}^{\left(l\right)}H_{k}^{T}+\frac{(\upsilon_{3}-2)\upsilon_{2}}{(\upsilon_{2}-2)\upsilon_{3}}R,\upsilon_{3}\right)=q_{k}^{\left(l\right)}\left(z\right),$$

so:

$$\begin{array}{ll} D_{k}(x)= & \left(1-p_{D,k})D_{k|k-1}(x\right)\\ & +\sum_{z\in Z_{k}}\sum_{j=1}^{J_{k|k-1}}\frac{p_{D,k}w_{k|k-1}^{\left(j\right)}q_{k}^{\left(j\right)}\left(z\right)St(x;m_{k|k}^{\left(j\right)}\prime (z),P_{k|k}^{\left(j\right)}\prime (z),\upsilon_{3}\prime )}{\kappa_{k}(z)+p_{D,k}\sum_{l=1}^{J_{k|k-1}}w_{k|k-1}^{\left(l\right)}q_{k}^{\left(l\right)}\left(z\right)}. \end{array}$$
